# Study of the Structure and Properties of Electrical Sand Concrete under Prolonged Exposure to Sulfate Environment

**DOI:** 10.3390/ma15238542

**Published:** 2022-11-30

**Authors:** Anastasiya Gordina, Aleksandr Gumenyuk, Irina Polyanskikh, Grigory Yakovlev, Igor Pudov

**Affiliations:** Department of Geotechnical Engineering and Building Materials, Kalashnikov Izhevsk State Technical University, Studencheskaya Str. 7, 426009 Izhevsk, Russia

**Keywords:** corrosion evaluation, sulfate attack, electrical conductivity, compressive strength, sulfate ingress, microstructure, leaching, microstructure

## Abstract

Destructive processes accompanying sulfate corrosion of concrete significantly affect the durability of products and structures based on Portland cement. In the presented study, the long-term effect of sulfate corrosion on the electrical properties of electrically conductive sand concrete was studied. In the course of the study, the following were tested: an electrically conductive composition and a control composition based on plain Portland cement. The analysis of changes in the mineral composition of the samples over the course of time in an aggressive solution was carried out. The results show that during the exposure period of the samples from 28 to 224 days, the absorption of sulfate ions slows down and averages 26% for the control composition and 29% for the electrically conductive composition, of the total volume of absorbed sulfates. At the same time, the course of sulfate corrosion was accompanied by a 6% increase in the density of samples of both compositions, as well as a cyclic change in mechanical strength within 15%. In its turn, the key indicator of the electrical characteristics of the compositions—electrical resistivity—tended to increase throughout the experiment. These results can be recommended for assessing the durability and the nature of the operating conditions of electrical concretes used in aggressive environments.

## 1. Introduction

Recent developments in the field of new multifunctional structural materials, such as “Smartconcrete”, led to a paradigm shift in the field of functional loading of building materials and structures towards products with additional functions of self-diagnostics, piezoresistive self-heating, as well as materials with the ability to control electrical properties. In the new context, traditional elements not only perform their basic function, but also include additional properties. For example, sidewalk and road surfaces integrate a resistive heating system for snow melting [1,2,3], increase the protective properties of concrete from electrochemical corrosion of reinforcement and form a negative potential at the mineral matrix [4], as well as install additional sensors for static [4] and dynamic [5] control of reinforced concrete structures [6] with the ability to detect cracks [4] and track dynamics of their changes.

The key indicator characterizing the possibility of giving an additional functional load to materials based on Portland cement is electrical resistivity [7,8]. This indicator is calculated, and today there are several methods for its determination [9]. The change in electrical resistivity is directly proportional to the change in the electrical conductivity of the material and depends on the chemical properties of the pore fluid and microstructure features.

For traditional concretes, the index ranges from 10^8^ Ωcm for samples dried to constant mass to about 10^2^ Ωcm for water-saturated samples [7]. In its turn, the maximum possible resistivity of electrically conductive concrete, which provides, for example, effective anti-icing application, is about 1000 Ωcm, which is two times lower than that of traditional concretes [3].

Progress in the use of carbon-containing materials, as well as the use of man-made waste to regulate the structure of mineral matrices, led to the emergence of a number of structuring additives used to ensure stable electrically conductive performance.

A review of existing studies shows that the first attempts to regulate the electrical properties of cement matrices were first described in [10,11]. There are three types of electrically conductive structures formed in the mineral matrix: cluster [12,13], linear [14], and complex [15]. To obtain each of the types, different types of additives are used: finely dispersed powders, predominantly round in shape, and particles with relative elongation [13,14,15]. Due to the peculiarities of the electrical conductivity [7] of the mineral matrix based on Portland cement, additives such as carbon-containing materials, fiber based on various types of metals, dispersed powders in the form of metal oxides or a mixture of doped oxides can be used to reduce the electrical resistivity [16,17]. When developing electrically conductive mineral matrices, attention is paid to the use of carbon-containing components of man-made origin due to their economic efficiency [18].

A significant number of experimental and theoretical studies indicate that the degree of electrical conductivity of a composite material based on Portland cement modified with electrically conductive additives is determined by reaching the percolation threshold [7,19]. Along with this, the factors [7,20] that have a direct impact on the electrical resistivity for a long time were studied. The main one is the recrystallization of crystalline hydrate and hydrosulfoaluminate compounds, which have clear dielectric properties at the level of 10^9^ Ωcm [7,20]. In view of this, it is proposed to use such additives as calcium chloride and calcium nitrite, containing the like-named cation with alite and belite, which ensures the acceleration of hydration processes [20,21]. The use of these additives leads to the rapid formation of CSH and reduces the intensity of ettringite formation [7,22], thus stabilizing the electrical resistivity. At the same time, a decrease in the degree of recrystallization stabilizes the characteristics of the pore space. Capillary suction forces, absorbing moisture from the environment, saturate the structure of the material with liquid and free ions, which also increases the electrical conductivity [18].

Analyzing the results of fundamental [5,22] and applied [23,24] studies on the use of electrically conductive concrete, we can talk about the need to use such materials in aggressive conditions and search for ways to increase durability.

Sulfate-containing environments are by far one of the most common of the potentially damaging impacts that reduce durability [25,26,27]. Sulfates are found in most natural waters and soils [28]. As analysis shows [28], in fresh water bodies, the concentration of sulfate ions is up to 60 g/l. At the same time, this indicator is much higher in mineralized groundwater. In turn, in sea water, the concentration of SO_4_^2−^ is from 2.5 to 2.7 g/L. This type of mineral matrix corrosion is characterized by certain destruction mechanisms, including chemical reactions between active sulfate ions and hydrates of cement stone, primarily tricalcium aluminate [26,28], accompanied by a physical impact caused by an increase in internal pressure due to recrystallization of new formations [22,27]. Some works consider both the general mechanisms of sulfate corrosion [29,30] and specific variants of its development, such as sulfate corrosion at low temperatures [31,32] or processes of delayed formation of ettringite [33,34]. The destructive effect of sulfate corrosion is the cause of numerous costly repairs and a significant number of structural failures [28,35]. Today, it is of interest to evaluate the features of the corrosion process not only for existing compositions and structures, but also for promising structural compositions and products. In this regard, the specific volume resistance of concrete is considered as one of the most important parameters, which is affected by sulfate corrosion. So, in the works, studies were carried out on the effect of sulfate corrosion on the electrical properties of mineral matrices during the first 28 days of hardening [5,22,36], without taking into account the features of the process during long service life.

To date, it is established that the rate of corrosion decreases with an increase in the specific resistance of concrete used under normal environmental conditions. At the same time, ambiguous results were obtained for structures used in a hydrosulfate environment [2,5,6,22]. The differences in the published results can be explained by the practiced methods of testing corrosion resistance, which are often carried out by exposing small samples to highly concentrated solutions of sulfates at elevated temperatures, which distorts the physicochemical processes, increasing the possibility of obtaining unreliable results [37,38,39]. It is common to use environments with a high concentration of aggressive substances (10% and more) as reagents with exposure to high temperatures (up to 80 °C), which far from always reflect the actual working conditions of structures. A number of authors [28,38] recommend observing the criteria for similarity of laboratory studies in the corrosion process to real conditions in order to reduce possible distortions of the results obtained.

In studies [30,32], the mechanism of sulfate corrosion of a mineral matrix is considered as a heterogeneous process. Previously [26,27], it was considered as a process with a constant diffusion coefficient in time, which does not correspond to reality due to the presence of deceleration processes, which require a detailed study of diffusion processes (diffusion control of the reaction). In turn, studies [36,40,41] show the need for parallel consideration of the conditions for the occurrence of sulfate corrosion not only in the area of transfer of reaction products from solution to the reaction active zone of the mineral matrix, but also the conditions for the transfer of reactants to the interface, which in turn is kinetic control. The mechanism of hydrosulfate corrosion is a multifactorial process, which is directly dependent on the conditions in which studies are carried out. To date, the most significant patterns of control of this process are established, such as temperature and concentration of sulfate ions. Thus, [32,39] the intensification of the process of sulfate corrosion can be achieved by regulating the heat–humidity regime. In studies [29,32], it was found that at an ambient temperature of up to 10 °C, the process of corrosion of set cement in a hydrosulfate environment proceeds more intensively than under normal conditions (air temperature 18–22 °C and relative air humidity 100%).

To simulate the corrosion process in laboratory conditions, a mode of changing aggressive solutions is required, which, to a certain extent, should take into account the decrease in the corrosion rate with time. In particular, it is obvious that on the first day of contact of the test samples with an aggressive environment, the interaction between them proceeds at a maximum speed; therefore, the concentration of ions in an aggressive environment decreases rapidly and is at a significantly lower level than expected during the experiment. For example, if one is testing the resistance of concrete in a solution containing 500 mg/L SO_4_^2−^, on the first day of the experiment, the concentration of the aggressive solution does not exceed 0.03–0.04% (300–400 mg/L SO_4_^2−^). With a sufficiently long test period, the concentration of the aggressive medium levels off and approaches the initial selected value, but this can only be achieved by periodically changing the solution. At the same time, an important aspect is the retention of the concentration of the aggressive environment during the first 28 days of testing by 1–2 orders of magnitude lower than the actual concentrations of the influencing media due to the high probability of distortion of the chemical mechanism of corrosion [28,29]. In this regard, the concentration range of the sodium sulfate solution is accepted in the range from 3 to 5% [30,31,32,33,34]. To improve the accuracy of the correspondence of physical and chemical processes to real conditions, it is recommended in [33,36,41] to use a 1% solution of sodium sulfate.

Sulfate corrosion today causes significant damage to the economies of countries around the world, and the fight against it requires huge material and technical costs. At the same time, at present, the problem of sulfate corrosion is not given sufficient attention. The flow process, quantitative indicators, and many other issues with the theory of sulfate corrosion of a mineral matrix based on Portland cement and methods for increasing the durability of structural elements of buildings and structures are yet to be sufficiently studied due to their duration and increased labor intensity, which necessitates the improvement of methods for testing the sulfate resistance of mineral matrices.

To date, there is a range of methods for accelerated testing of traditional concrete for corrosion resistance [28,39]. In the review study [42], the main methods are distinguished, such as: the method of constant exposure to a hydrosulfate medium [30,38], the method of cyclic saturation and drying [32,39], the method of stochastic dynamic reactions of nonlinear systems [38], potentiometric method [40,41], methods of Moskvin and Kind [27], etc. The potentiometric method of analysis has significant advantages over its analogues due to the possibility of a phased study of the ettringite–thaumasite system [40,43].

Thus, the paper presents the results of a study obtained by the potentiometric method, which includes the constant effect of a hydrosulfate medium on the mineral matrix. The results of the influence of the long-term impact of sulfate corrosion on the electrical and mechanical properties, as well as on the change in the microstructure at the later stages of the formation of the mineral matrix, are presented.

## 2. Materials and Methods

In the presented study, the compositions of sand concrete are analyzed: a control composition and a composition with an electrically conductive additive introduced in the form of a suspension.

**Figure 1:**
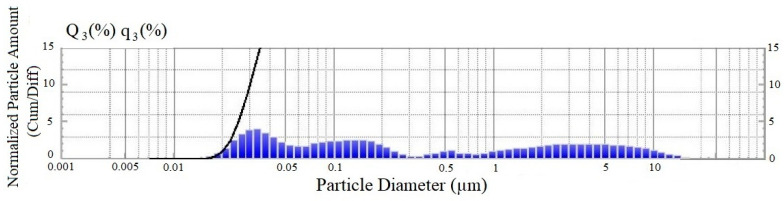
Granulometric composition of the dispersed phase of the electrically conductive additive.

Suspension of industrial soot UPC-MIX-1, produced by Novy Dom LLC, is a pigment concentrate, including industrial soot in the amount of 32%. Solid particle size distribution is measured with a laser diffraction analyzer (Figure 1 and Table 1). An analysis of the distribution of particles showed that the additive is characterized by a poly-sized dispersed phase, represented by micro- and nano-particles in a wide range from 0.03 to 10 microns.

For the manufacture of the compositions, the following were used: Portland cement CEM I 42.5N produced by Novoroscement, OJSC. The chemical composition of cement is a set of components in the following percentage: CaO—66.73%; SiO_2_—23.22%; Al_2_O_3_—5.16%; Fe_2_O_3_—4.42%; and SO_3_—0.47%. In turn, the mineralogical composition of cement is also represented by components in the ratio: C_3_S—65%; C_2_S—13%; C_3_A—4%; and C_4_AF—18%. Graded quartz sand with fineness modulus Mf = 1.25 was used as a filler, meeting the requirements of GOST 8736-2014; mixing liquid corresponding to GOST 23732-2011.

In turn, to stabilize the electrically conductive properties, calcium nitrate was introduced into the mixture corresponding to GOST 4142-77 “Calcium nitrate 4-hydrate”, which was previously dissolved in mixing water.

Table 2 shows the ratio of materials used to make experimental mixtures. At the same time, the effective percentage of the applied polyfunctional additives was taken on the basis of previous studies [36,40].

### 2.1. Experimental Process

#### 2.1.1. Exposure

The potentiometric method was used to study the chemical resistance and evaluate the effect of an aggressive sulfate medium on the properties of the compositions. This method includes recreating hydrosulfate corrosion conditions using a 1% Na_2_SO_4_ solution, as well as performing a titrimetric analysis of the solution at control times to assess the rate of absorption of SO_4_^2−^ ions by samples and determine the volume of released calcium hydroxide. In turn, the concentration of ions in the solution is:

M (Na_2_SO_4_ × 10H_2_O) = [2 × 23 + 32 + 4 × 16 + 10 × (2 + 16)] = 322 g/mol

M (SO_4_^2−^) = 96.06 g/mol

M SO_4_^2−^/M Na_2_SO_4_ × 10H_2_O = 96.06/322 = 0.298 = 0.3

1000 × 0.3 = 300 mg. (SO_4_^2−^)/L.

The method of preparing the solution is as follows: in 1 L of distillate at a temperature of 20 ± 1 °C, Na_2_SO_4_ × 10H_2_O powder was poured out and stirred in a laboratory vessel for 3–5 min until the salt was completely dissolved.

Next, cube samples of 20 × 20 × 20 and 70 × 70 × 70 mm were placed in the obtained solution in a desiccator, which were kept in the solution for 224 days, with control measurements carried out on days 56, 112, and 224 of exposure [26,27].

To maintain the concentration of sulfate ions in the solution, before titration, the pH was determined on an ST5000-F pH meter (with an ST350 electrode), after which, the solution was titrated with a 5% H_2_SO_4_ solution to restore the conditions of an N sulfate medium. Titration was carried out until the color of the indicator changed from crimson to neutral white, after which, the pH was determined. Titration was carried out every day for the first 7 days, every two days until day 14, every three days until day 28, and every 7 days until day 224.

When the control period was reached, the samples were removed from the solution and subjected to strength tests, mass measurements, electrical resistance measurements, and were examined by a set of physical and chemical analysis tools.

#### 2.1.2. Test Methods and Preparation of Specimens

##### Specimens

The program of the presented study included the preparation of samples of the control and electrically conductive composition; for each composition, samples were made from dough of normal density with dimensions of 70 × 70 × 70 mm and 20 × 20 × 20 mm. Two days after molding, the samples were removed from the mold and placed in water at 20 ± °C until brand strength was reached. Then the samples were removed and placed in the prepared solution. The samples with dimensions of 70 × 70 × 70 mm were used to evaluate the strength and electrical properties.

For the correlation analysis of the relationship between the mechanical compressive strength and the volume of released calcium hydroxide and the assessment of the physicochemical properties, the samples of 20 × 20 × 20 mm were used, and these types of samples were also used to determine the change in mass.

The combination of small sample sizes and the use of an N sodium sulfate solution made it possible to carry out studies under conditions close to real ones [26,28].

##### Testing Methods

*Compressive Strength of Concrete*

The compressive strength of the samples was determined at the age of 56, 112, and 224 days using a PGM-100MG4-A hydraulic press with a loading rate of 0.5–0.8 MPa/s.

*Electrical Resistance Measurement Test Methods*

To obtain data for calculating the specific volume resistance of mineral matrices, the probe method was used. The probe method is the introduction of two pin-type copper probes into the sample at a given distance from each other [3,36].

The MNIPI E7-20 device was used to measure current voltage characteristics. The principle of operation of the device is based on the voltmeter–ammeter method. The operating frequency voltage from the generator is supplied through the measured object to the converter, which generates two sinusoidal voltages (proportional to the current flowing through the object and proportional to the voltage on the object), which are converted into digital form. The value of the measured parameters is determined by calculation and displayed on the display in Ohm, Ω.

At the same time, based on the data obtained from the device and the geometric parameters of the samples, the electrical resistivity was calculated using the formula [3,36]:

ρ = R·A/L, Ω·cm,

where ρ is sample resistivity; R—sample resistance; L—distance between probes; and A—sample cross-sectional area.

*Microstructure Examination of Concrete*

To study the morphological features, chips of samples were taken after 56, 112, and 224 days of exposure in an aggressive environment. The features of the microstructure of the studied composite materials were determined using the scanning electron microscopes Thermo Fisher Scientific Quattro S (Center for Collective Use “Center for Physical and Physicochemical Methods of Analysis, Study of the Properties and Characteristics of Surfaces, Nanostructures, Materials and Products” UdmFITSUrO RAS) and Tescan Mira3 XMU. The Thermo Fisher Scientific Quattro S survey was carried out in low vacuum mode from 20 to 30 kV, without deposition, at a pressure of 50 Pa, with ×5000 magnification steps, while the Tescan Mira3 XMU survey was carried out at 20 kV, with gold sputtering.

*Thermogravimetry–Dierential Scanning Calorimetry (TG–DSC) Analysis of Concrete*

DSC analysis was used to obtain additional information on the phase composition of the studied materials. DSC data were obtained on a TGA/DSC1 instrument from Mettler-Toledo Vostok CJSC in a temperature range from 60 to 1100 °C and at a temperature rise rate of 30 °C/min in air.

## 3. Results

### 3.1. Corrosion Resistance

The study of the process of sulfate corrosion was carried out in a significantly longer period from 56 to 224 days of exposure and is a continuation of work [36,40,44]. The obtained experimental results characterize the features of the modified and unmodified mineral matrix.

**Figure 2:**
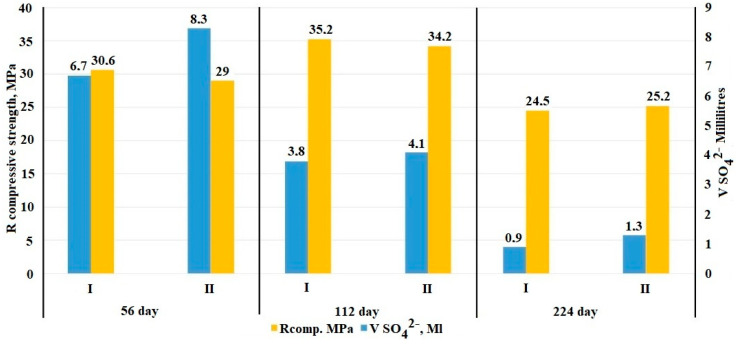
Correlation of data on the volume of absorbed SO_4_^2–^ ions from a 1 N aqueous solution of sodium sulfate by samples of control and electrically conductive concrete with a change in strength characteristics (Note to Figure 1. The value of the coefficient of variation was V = 6.7% (control composition—I) and V= 7.1% (electrically conductive composition—II).

The graph presented in Figure 2 demonstrates the process of the influence of 1 N sodium sulfate solution on the strength characteristics of the studied compositions and their change over time.

The analysis of the results of the titrimetric study was carried out up to 224 days. The period was determined on the basis of the works [26,27] and is optimal for passing the periods of kinetic, mixed, and diffusion control.

At the initial stage of research on the 28th day of exposure (detailed results are presented in [36]), the control composition absorbed 31.6 mL of SO_4_^2−^ and the electrically conductive composition—32.6 mL SO_4_^2−^. In turn, for the remaining period, the absorbed volume was 11.4 mL of SO_4_^2−^ for the control composition and the electrically conductive composition was 13.7 mL of SO_4_^2−^. Thus, the total volume of absorbed SO_4_^2-^ for the entire test period was for the control composition—43.1 mL; and for the electrically conductive composition—46.3 mL.

On the 224th day of testing, the reactive components of the mineral matrix completely reacted with sulfate ions in the area of the samples, with a depth of 4.5 mm up to 2 cm from the surface, which indicates the transition of the reaction to the diffusion stage.

The obtained data on titration and mechanical strength at an early stage of the study (up to 28 days) indicate an active growth of the expansive phase, while due to the filling of micro and macropores, an increase in the density of the samples was noted, which is confirmed by the strength and mass indicators. At the next stage of the study (from 28 to 224 days), a stable growth of the expansive phase was observed, confirmed by an increase in the mass of the samples; the data are presented in Table 3, while the mechanical strength indicators indicate the filling of the internal space and an increase in internal stress, which results in a drop of the strength of both compositions by an average of 35% on the 224th day.

In turn, Table 3 shows data from which it follows that the mass of the samples increases on days 56 and 112, which is caused by mixed diffusion kinetic processes due to the penetration of sulfate ions. The change in mass on the 224th day is possible due to recrystallization and compaction of the near-surface structure. The effect of the solution at this stage is manifested due to the diffusion of ions.

### 3.2. Electrical Properties

**Figure 3:**
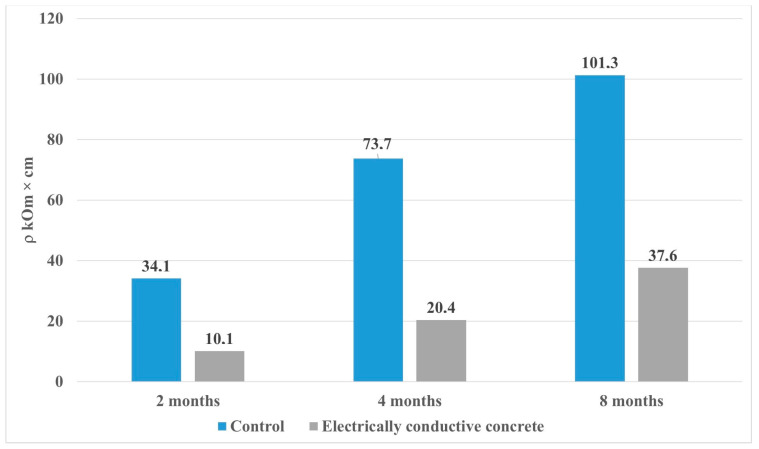
Effect of hydrosulfate corrosion on the electrical resistivity of samples.

The results of the study of the effect of hydrosulfate corrosion on the electrical properties of the control and electrically conductive compositions, shown in Figure 3, show that due to the intensive growth of the expansive phase with pronounced dielectric properties [7] there is a regular increase in the specific volumetric electrical resistance of the samples to the level of the dielectric. One should note the difference in resistivity between the electrically conductive composition and the control composition, which is 65%, and is provided by the achievement of the percolation threshold, through the presence of a cluster structure of electrically conductive components in the modified composition.

### 3.3. Microstructural Morphology by SEM

**Figure 4:**
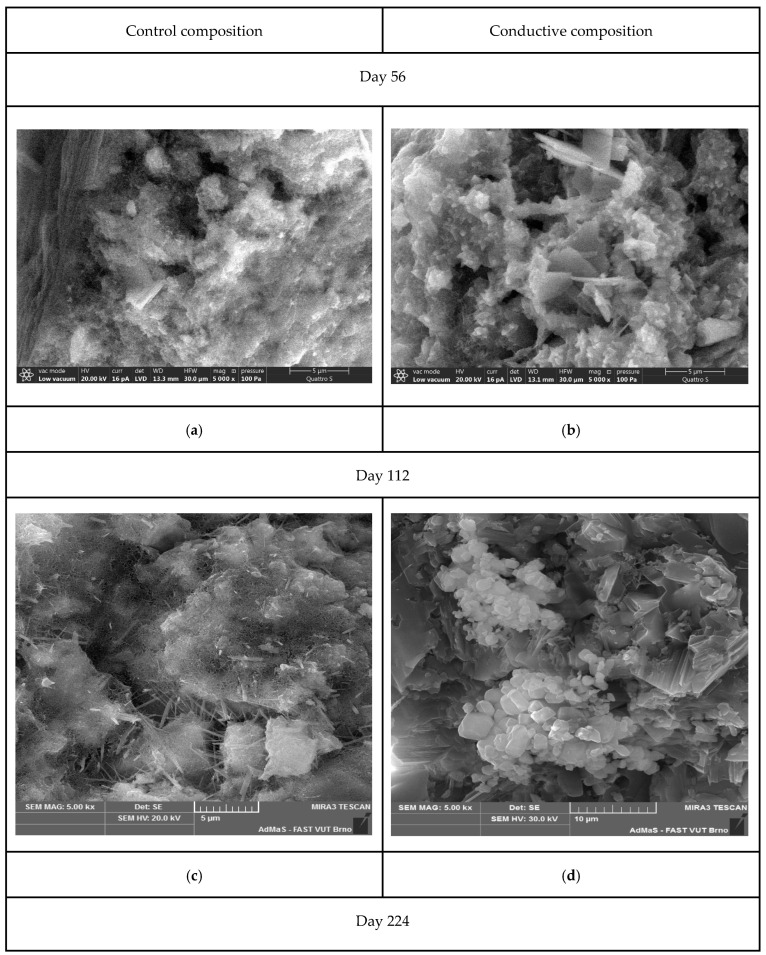

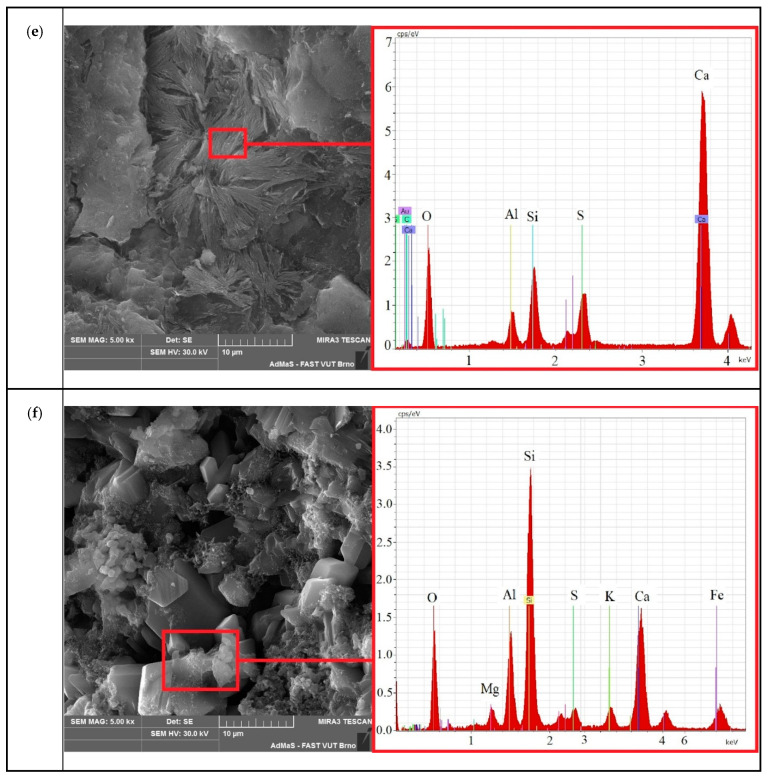
Scanning electron microscopy of samples of the control (**a**,**c**,**e**) and electrically conductive compositions on (**b**,**d**,**f**): (I) day 56 (**a**,**b**); (II) day 112 (**c**,**d**); and (III) day 224 (**e**,**f**).

During the impact of sulfate ions on the mineral matrix, the structure of the samples underwent a number of physicochemical changes (Figure 4). An extended study of the microstructure made it possible to establish the nature and change in new formations in the compositions.

The resulting images visualize the surface morphology of the cleavages of the control and electrically conductive compositions. The structure of the control sample (a) on the 56th day of testing is a polymineral aggregate of set cement, including voids formed during the washing out of calcium hydroxide. Analysis of the morphology of the electrically conductive composition (b) is characterized by the formation of crystalline hydrates of various natures, including tabular calcium hydroaluminates.

On the microstructure of the samples after 112 days of testing, the following changes can be observed: (c)—in the structure of the control sample, the formation of microcracks caused by the formation of secondary ettringite (acicular crystalline hydrates) is observed; (d)—the structure of the electrically conductive composition is represented by formations of cubic calcium hydroferrites, as well as polymineral conglomerates of set cement.

On the 224th day in the structure of the control sample—(e) the splitting of acicular crystals of ettringite to fibrous crystals with the formation of “two-leaf” is observed; in electron microscopic images of electrically conductive concrete—(f) crystalline hydrates of set cement are observed, including electrical calcium sulfoaluminoferrites in the form of hexagonal crystals and an amorphous phase.

A decrease in the concentration of calcium hydroxide, due to its leaching at an early stage of corrosion, at later stages makes it impossible for the formation and existence of polybasic calcium hydroaluminates, which, in turn, prevents and sometimes excludes the possibility of the formation of calcium hydrosulfoaluminates. At a later stage, during the action of sodium sulfate, along with the formation of gypsum, accumulations of well-defined spherulites of calcium hydroaluminate and calcite are presented in a slightly larger amount.

### 3.4. Differential Thermal Analysis of Compositions

**Figure 5:**
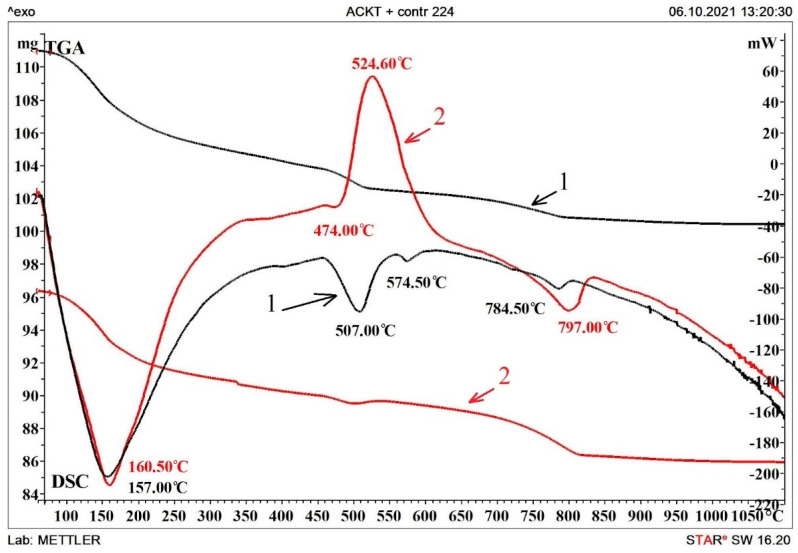
DSC analysis of samples on day 224 of the test: 1—control composition; 2—electrically conductive composition.

To establish the relationship and interpret the causes of changes in physical and mechanical parameters, differential scanning calorimetry was carried out in an oxygen atmosphere. The curves obtained are shown in Figure 5.

Figure 5 and Table 4 show the temperature curves and data on the weight loss of the samples upon heating. A comparative analysis found that in the range from 90 to 400 °C, a peak of 160.5 °C for the electrically conductive composition is associated with dehydration of set cement hydration products, including monosulfoaluminate. At the same time, the peak of 157.0 °C in the control composition is associated only with the dehydration of the gel and crystalline products of cement hydration. The presence of this peak in the region from 100 °C to 300 °C, as well as the shoulder of the graph at about 300 °C, indicates the presence of ettringite in the compositions. The endothermic effect in the region of 495–520 °C and 507 °C on the graph of the control composition can also be attributed to the thermal decomposition of ettringite and calcium hydroxide. At the same time, the temperature effect and weight loss in the temperature range of 775–840 °C for the control sample—784.5 °C/−1.6% and for the electrically conductive sample 797 °C/−3.7% are associated with the decomposition of mettringite, carbonates, and calcium aluminoferites. A large loss of mass of the electrically conductive composition is possibly due to a large amount of ettringite in the composition [43,45].

## 4. Conclusions

Experimental data obtained by analyzing the dynamics of the process of absorption of sulfate ions, microstructure, thermal effects for samples of control, and electrically conductive compositions show the features of the long-term effect of an N sodium sulfate solution on the characteristics of fine-grained concrete under the external influence of sulfates. Based on the laboratory results, the following conclusions can be drawn:

1. The mode of constant exposure to an N sodium sulfate solution at the later stages of the experiment showed that in the period from 28 to 224 days, the absorption of sulfate ions slows down and averages 26% for the control and 29% for the electrically conductive compositions of the total volume of absorbed sulfates.
2. Monitoring of the physico-mechanical parameters of the samples during the entire time of exposure to a 1 N sodium sulfate solution showed an increase in the density of the samples, for the control composition—by 6%, for the electrically conductive one—by 6.5%. In turn, the cyclic change in the mechanical strength, on average by 15% for the control and electrically conductive samples, in the period from 56 to 224 days, is a relaxing release of internal stress, followed by compaction of the formed microcracks.
3. The electrical resistivity of the samples has a steady growth trend during the entire experiment, while the growth in the control and electrically conductive compositions has a different character. Thus, the increase in the resistivity of the control composition for the period from 28 to 224 days was 75%, for the electrically conductive composition of 74%, while the final value is 101.3 kOhm cm for the control sample, and for the electrically conductive composition is 37.6 kOhm cm, which indicates a difference in the physicochemical properties of the phase composition of mineral matrices.
4. An analysis of the microstructure of the samples showed that in the control composition, at the later stages of the experiment (from 56 to 224 days), ettringite splits with the formation of “two-leaves”. At the same time, the formation of calcite spherulites and calcium hydroaluminates is observed in the electrically conductive composition, which leads to an increase in the amount of the solid phase, to a cyclic increase in crystallization pressure, and causes a negative effect in the form of a decrease in strength.
5. Differential thermal analysis confirmed the differences between the features of the interaction of compositions with an aggressive environment and a decrease in strength characteristics.

The obtained results of the research can be used in preparing recommendations for the development and optimization of the compositions of electrically conductive concrete used in aggressive conditions of hydrosulfate corrosion, it might also be of great importance for choosing the conditions for conducting experimental studies on the process of sulfate corrosion of composite compositions based on Portland cement.

## Figures and Tables

**Table 1:** Distribution parameters of the granulometric composition of the electrically conductive additive, in % by weight.

Particle Size, Microns	Content, %
0.014–0.091	34.8
0.1–1.05	28.3
1.05–20	36.9

**Table 2:** Component ratio of compositions.

Mix	Portland Cement, CEM I 42.5 g	Quartz Sand, g	Industrial Soot, %	Calcium Nitrate %	Water-Cement Ratio [29]
Control	800	1600	-	-	0.5
Electrically conductive concrete	-	-	7	3	

**Table 3:** Change in the mass and density of the samples during the test from the initial value.

Curing Period, Days	56		112		224	
Values	m	ρ	m	ρ	m	ρ
Control	+5%	+3%	+2%	+2%	+1%	+1%
Electrically conductive composition	+3%	+3%	+1.5%	+1.5%	+2%	+2%

**Table 4:** Temperature effects of control and electrically conductive composition.

Temperature Range	Effect Temperature/Mass Loss		Effect
	Control Composition (1)	Electrically Conductive Composition (2)	
100–300 °C	157.0/−5.6%	160.5/−5.65%	Dehydration of gel and crystalline set cement products, including calcium. monosulfoaluminate
500–800 °C	-	524.6/−1.6%	Oxidation of the carbon component.
495–520 °C	507.00; 574.5/−2.3%	-	Decomposition of elements of the ettringite system and recrystallization of silicon oxide.
775–840 °C	784.5/−1.6%	797.0/−3.7%	Dehydration of ettringite, calcium carbonates, and hydroalumoferites.
